# Drug-related harm among people who inject drugs in Thailand: summary findings from the Mitsampan Community Research Project

**DOI:** 10.1186/1477-7517-10-21

**Published:** 2013-10-07

**Authors:** Kanna Hayashi, Lianping Ti, Nadia Fairbairn, Karyn Kaplan, Paisan Suwannawong, Evan Wood, Thomas Kerr

**Affiliations:** 1 Urban Health Research Initiative, British Columbia Centre for Excellence in HIV/AIDS, St. Paul’s Hospital, 608-1081 Burrard Street, Vancouver, BC V6Z 1Y6, Canada; 2 Interdisciplinary Studies Graduate Program, University of British Columbia, Green College, Green Commons, Room 153A, 6201 Cecil Green Park Rd, Vancouver, BC V6T 1Z1, Canada; 3 Faculty of Medicine, University of British Columbia, 317-2194 Health Sciences Mall, Vancouver, BC V6T 1Z3, Canada; 4 Mitsampan Harm Reduction Center / Thai AIDS Treatment Action Group, 18/89 Vipawadee Rd., soi 40 Chatuchak, Bangkok 10900, Thailand

**Keywords:** Injection drug use, Drug law enforcement, Harm reduction, Community-based participatory research, Thailand

## Abstract

**Background:**

For decades, Thailand has experienced high rates of illicit drug use and related harms. In response, the Thai government has relied on drug law enforcement to address this problem. Despite these efforts, high rates of drug use persist, and Thailand has been contending with an enduring epidemic of human immunodeficiency virus (HIV) among people who inject drugs (IDU).

**Methods:**

In response to concerns regarding drug-related harm in Thailand and a lack of research focused on the experiences and needs of Thai IDU, the Mitsampan Community Research Project was launched in 2008. The project involved administering surveys capturing a range of behavioral and other data to community-recruited IDU in Bangkok in 2008 and 2009.

**Results:**

In total, 468 IDU in Bangkok were enrolled in the project. Results revealed high rates of midazolam injection, non-fatal overdose and incarceration. Syringe sharing remained widespread among this population, driven primarily by problems with access to syringes and methamphetamine injection. As well, reports of police abuse were common and found to be associated with high-risk behavior. Problems with access to evidence-based drug treatment and HIV prevention programs were also documented. Although compulsory drug detention centers are widely used in Thailand, data suggested that these centers have little impact on drug use behaviors among IDU in Bangkok.

**Conclusions:**

The findings from this project highlight many ongoing health and social problems related to illicit drug use and drug policies in Bangkok. They also suggest that the emphasis on criminal justice approaches has resulted in human rights violations at the hands of police, and harms associated with compulsory drug detention and incarceration. Collectively, the findings indicate the urgent need for the implementation of evidence-based policies and programs in this setting.

## Background

Thailand has experienced longstanding epidemics of illicit drug use and human immunodeficiency virus (HIV) among people who inject drugs (IDU). During the 1970s, Thailand became the world’s largest opium refining and distribution center, and accordingly, heroin use quickly became a major driver of drug-related harm in this setting [1,2]. Since the late 1990s, there has been a dramatic increase in the use of methamphetamine in Thailand, which has become the most commonly used illicit drug in the country today [3]. In response, the Thai government has relied on criminal justice approaches in an effort to eradicate illicit drugs. However, the national household survey indicates that illicit drug use remains widespread, with an estimated over 5% of the population having used illicit drugs in 2007 [4]. The prevalence of HIV among Thai IDU remains strikingly high, with approximately 30-40% of IDU living with HIV during the past two decades [5]. Although the Thai government offers a range of HIV prevention, care and treatment services for free (e.g., HIV testing and antiretroviral therapy), past reports suggested that IDU faced many barriers to access these services [6].

In response, IDU in Thailand have organized themselves and called for funding to institute evidence-based harm reduction and treatment strategies for them. In 2003, a drug user-driven harm reduction initiative was launched with funding from the Global Fund to Fight to AIDS, Tuberculosis and Malaria (GFATM) [7]. Although this civil society-driven movement led to the inclusion of methadone treatment in the national health security program in 2008 [5], needle and syringe programs (NSPs), which is recommended by the World Health Organization (WHO) and other United Nations (UN) agencies as an essential HIV prevention service for IDU [8], remain controversial: While public health authorities have endorsed NSPs, legal authorities regard it as illegal [9,10]. The legal uncertainty has created a challenging environment for some civil society organizations that were allowed to operate NSPs with funding from the GFATM, while their service providers were routinely arrested by police [6,11]. To date, the coverage of NSPs remains as low as less than 1% among Thai IDU [12].

In 2002, Thailand enacted the Narcotic Addict Rehabilitation Act B.E. 2545, which reclassified people who use drugs as “patients” instead of “criminals.” Despite this reclassification, in practice, Thailand continues to support large-scale police crackdowns and the expansion of compulsory drug detention centers (CDDCs) [13,14]. Most notably, a “war on drugs” policy in 2003 led to the extrajudicial killings of over 2,800 people and sparked criticism both domestically and internationally [15,16]. However, the intensive drug law enforcement-based approach continues to be endorsed by successive Thai governments [17,18], and the impact of this policy approach on the health and behavior of IDU remains unevaluated. As well, little is known about the coverage, quality, and effectiveness of public health programs for IDU in this setting.

In light of persistent concerns regarding drug policy in Thailand, the Mitsampan Community Research Project, an academic-community research partnership involving people who use drugs in Bangkok, was launched in 2008. This report briefly describes the project and summarizes the key peer-reviewed findings from the project.

## Mitsampan Community Research Project and research methods

The Mitsampan Community Research Project is a collaborative research effort involving the Mitsampan Harm Reduction Center (MSHRC; Bangkok, Thailand), Thai AIDS Treatment Action Group (TTAG; Bangkok, Thailand), Chulalongkorn University (Bangkok, Thailand), and the Urban Health Research Initiative of the British Columbia Centre for Excellence in HIV/AIDS/University of British Columbia (Vancouver, Canada). The MSHRC is a drug user-run drop-in center opened in 2004 with funding from the GFATM. The center provides a range of programs and harm reduction services (e.g., NSPs, health education, counseling and assistance in accessing healthcare), and is also active in advocating for the human rights of people who use drugs. The overarching objectives of this research were to investigate patterns of drug use, health services use, interactions with the criminal justice system, and health-related harms among IDU in Bangkok.

The specific methods employed in this research have been described in detail elsewhere [19]. In brief, it employs a serial cross-sectional study design, and the data used for this report were collected over two cycles of surveying in August 2008 and June – July 2009. The study participants were all active IDU residing in Bangkok or in adjacent provinces and being ≥18 years old when they enrolled in the study. Active IDU were defined as individuals who had injected drugs at least once in the six months prior to the interview. In 2008 and 2009, potential study participants were contacted through peer outreach and word-of-mouth, and were invited to the MSHRC to participate in the study. After providing oral informed consent, participants completed an interviewer-administered questionnaire covering a range of topics including demographics, drug use patterns, HIV risk behavior, health problems, access to healthcare and harm reduction services, and experiences with the criminal justice system. HIV seropositivity was also determined through self-reporting. Since the study’s focus on vulnerable populations and collecting data on illegal activities raised ethical concerns, we employed oral consent to protect the participant’s anonymity and confidentiality. Additionally, the data collected did not include any identifying information or permit identification of specific individuals. The study was approved by the research ethics boards of Chulalongkorn University and the University of British Columbia.

This report summarizes the results of twelve peer-reviewed studies conducted through the Mitsampan Community Research Project. In keeping with the overarching research objectives, four studies were conducted to investigate four different aspects of harms associated with drug use [20-23]. Similarly, four studies examined experiences of four different dimensions of drug law enforcement [24-27], and four studies focused on access to various healthcare and harm reduction services among IDU in Bangkok [28-31]. Because each study addressed a different research question, they employed different participant eligibility criteria, variables, sample sizes, and statistical analyses. The analytical methods have been described in detail in each published study. In most of the studies, conventional regression methods (i.e., multivariable logistic regression) were used to address the study objective.

## Results: summary of findings

### IDU recruitment

In total, 252 IDU participated in the study in August 2008, and 317 IDU participated in the study between June and July 2009. Therefore, an accumulated total of 569 IDU were enrolled in the study over the two years. Because 101 individuals participated in the study in both years, the study reached a total of 468 unique IDU (252 individuals in 2008 and 216 individuals in 2009). Sample characteristics are shown in Table 1. As shown, 157 (27.6%) were women, and the median age was 36 years (interquartile range [IQR]: 32 – 46 years). The three most commonly injected drugs by the study participants during the previous six months were: heroin (91.3%), midazolam (65.9%), and methamphetamine (57.5%) in 2008; and midazolam (80.4%), heroin (65.3%), and methamphetamine (65.3%) in 2009. The prevalence of self-reported HIV seropositivity was 17.4% over the two years. Of note, 362 IDU (77.4%) newly accessed the MSHRC as a result of their participation in the study, and an increase in the attendance rate at the MSHRC has been observed since the launch of the project [19]. The effective involvement of MSHRC members in the study likely facilitated the observed high rates of IDU recruitment [19].

**Table 1:** **Characteristics of a community-recruited sample of IDUs in Bangkok, Thailand, participating in the Mitsampan Community Research Project in 2008 and 2009 (*n*** = **569)**

**Characteristic**	**Total *n *(%)**	**Study enrolment**	
		**2008**	**2009**
		**252 (44.3%)**	**317 (55.7%)**
		***n *(column%)**	***n *(column%)**
Female gender	157 (27.6%)	66 (26.2%)	91 (28.7%)
Age			
< 35 years	243 (42.7%)	111 (44.0%)	132 (41.6%)
35-45 years	175 (30.8%)	74 (29.4%)	101 (31.9%)
≥ 46 years	151 (26.5%)	67 (26.6%)	84 (26.5%)
Drugs injected at least once^a^:			
Heroin	437 (76.8%)	230 (91.3%)	207 (65.3%)
Methamphetamine	352 (61.9%)	145 (57.5%)	207 (65.3%)
Midazolam	421 (74.0%)	166 (65.9%)	255 (80.4%)
Self-reported HIV seropositivity	99 (17.4%)	29 (11.5%)	70 (22.1%)
Being on antiretroviral therapy^b^	54 (54.5%)^c^	21 (72.4%)^c^	33 (47.1%)^c^

IDUs: injection drug users.

^a^ Denotes activities during the 6 months prior to the interview.

^b^ Denotes activities at the time of interview.

^c^ The number of HIV-infected individuals is used as a denominator.

### Drug-related harm

In order to better understand the health status of and risks facing IDU in Bangkok, four studies were conducted to examine the prevalence and correlates of drug-related harm commonly experienced among the study participants. Anecdotal reports suggested that an increasing number of IDU in Bangkok were injecting midazolam—a short-acting benzodiazepine that can be acquired through private clinics. In 2008, over two thirds (67.5%) of participants in our study reported a history of midazolam injection, and 57.1% reported daily injection of midazolam in the previous six months. Midazolam injection was independently associated with poly-substance use (adjusted odds ratio [AOR] = 5.86; 95% CI: 2.96 – 11.60) and binge drug use (AOR = 2.25; 95% CI: 1.09 – 4.63), and was commonly used in combination with both opiates and methamphetamine [20].

**Figure 1:**
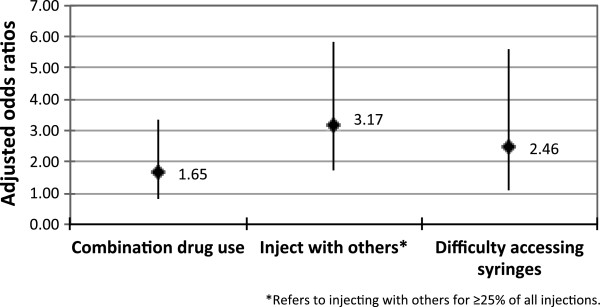
**Multivariate logistic regression analysis of factors associated with syringe borrowing among Thai IDU (*n*** = **238).**

Our study involving 238 IDU in 2008 demonstrated that 30.3% of participants reported syringe borrowing in the past six months. Consistent with past research [32,33], syringe borrowing was defined as injecting with a syringe used by others. As shown in Figure 1, syringe borrowing was independently associated with difficulty accessing sterile syringes (adjusted odds ratio [AOR] = 2.46; 95% confidence interval [CI]: 1.08–5.60). Primary reasons for experiencing difficulty accessing syringes included being too far from syringe outlets, pharmacies being closed, and being refused syringes at pharmacies [21]. These findings suggest that poor access to sterile syringes is driving the high rate of syringe borrowing observed in this study, and various lines of evidence corroborate this interpretation [34,35].

Methamphetamine injection appeared to be increasing among IDU in Bangkok, raising concerns about the associated impacts on HIV risk behavior among this population. Among 311 study participants in 2009, 36.7% reported having injected methamphetamine pills, locally referred to as “*yaba,*” twice or more per week in the previous six months. The prevalence of methamphetamine injection observed in this study was much higher than previously reported rates using data collected during 1999 and 2004 [36,37]. In multivariate logistic regression analyses, after adjustment for potential social, demographic and behavioral confounders, syringe sharing (i.e., borrowing or lending used syringes from or to others) in the past six months remained independently associated with injecting methamphetamine more than once per week (AOR = 2.86; 95% CI: 1.59–5.15) [22].

Although drug-related overdose is a leading cause of death among people who use drugs globally [38-40], the overdose experiences of Thai IDU have not been investigated. Our study conducted in 2008 found that 29.8% of participants had a history of overdose, and reporting a history of overdose was independently associated with a history of incarceration (AOR = 3.83; 95% CI: 1.52 – 9.65). The majority of participants (67.9%) had also responded at the scene of an overdose. While many reported responses with resuscitative potential, almost half reported engaging in responses with low life-saving potential, such as injecting the individual with salt water [23].

### Experiences with drug law enforcement

Given the ongoing emphasis on drug law enforcement in Thailand, we conducted a series of analyses to examine experiences with drug law enforcement among 252 IDU recruited in 2008. The first study exploring incarceration experiences demonstrated high rates of HIV risk behavior among IDU who had been in prison. The majority of participants (78.2%) reported a history of incarceration, and approximately 30% reported using drugs while in prison; 81.4% of these individuals also shared used syringes while incarcerated. A history of imprisonment was independently associated with a history of syringe sharing (AOR = 2.20; 95% CI: 1.12 - 4.32) [24].

Our examination of the prevalence and correlates of experiences with CDDCs revealed that 31.7% of participants had been in a CDDC at some point. As shown in Table 2, the exposure to CDDCs was independently associated with experiencing police abuse (i.e., having illicit drugs planted on oneself by police) (AOR = 1.81; 95% CI: 1.04 – 3.15). Further, the intensity of recent injection drug use did not differ between those who were and were not exposed to CDDCs (*p* > 0.14) [25]. These findings suggest that CDDCs are associated with police abuse and appear not to be helping to reduce drug use among IDU in Bangkok.

**Table 2:** **Multivariate logistic regression analysis of factors associated with compulsory drug detention exposure among Thai IDU (*n *= 252)**

**Variable**	**Adjusted Odds Ratio (AOR)**	**95% Confidence Interval (CI)**	***p*-value**
**Ever used drugs in combination**			
(yes vs. no)	1.78	(0.94 – 3.36)	0.078
**Ever had drugs planted by police**			
(yes vs. no)	1.81	(1.04 – 3.15)	0.035
**Median daily expenses for purchasing drugs**			
(≥ 300 THB vs. < 300 THB)	1.86	(1.07 – 3.22)	0.028

Reproduced from Csete et al. [25].

Previous studies have shown that intensive drug market policing can produce harmful impacts on public health [41]. In two separate studies, encounters with police were operationalized into two variables: (1) experiences with evidence planting by police (in the form of police placing illicit drugs on an individual) as an indicator of direct encounters with police; and (2) perceiving an increase in police presence where people obtained or used drugs in the previous six months as an indicator of indirect encounters with police. The first study revealed a high rate of police abuse against IDU: 48.4% of participants reported having illicit drugs planted on them by police. In multivariate analyses, this form of police misconduct was strongly associated with a history of overdose (AOR = 2.56; 95% CI: 1.40 – 4.66), syringe lending (AOR = 2.08; 95% CI: 1.19 – 3.66) and having been in a CDDC (AOR = 1.88; 95% CI: 1.05 – 3.36). Moreover, among those who reported having drugs planted on them by police, almost half (48.3%) paid police a bribe (median = 5,000 Thai Baht or approximately $140 USD) in order to avoid arrest [26].

Among the same sample, 54.4% reported observing an increase in police presence where they obtained or used drugs in the previous six months. However, levels of drug use did not vary among those who did and did not report observing an increase in police presence [27]. Despite the continued use of police crackdowns, the findings suggest that increasing police presence in drug markets appears to have had little effect in reducing drug use among IDU in Bangkok.

### Access to health care and harm reduction services

**Figure 2:**
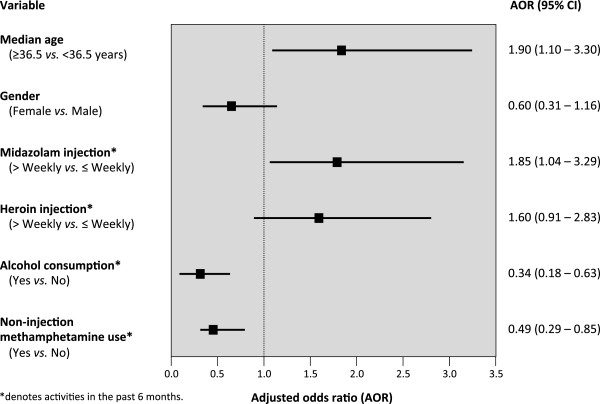
**Multivariate logistic regression analysis of factors associated with accessing methadone treatment in the previous 6 months among a community-recruited sample of people who inject drugs in Bangkok, Thailand (*n*** = **273).**

Four studies have been conducted to examine access to essential health and harm reduction services among IDU in Bangkok. The first study sought to investigate access to methadone treatment, one of the core interventions for HIV prevention and care for IDU [8]. While methadone treatment has been available in Bangkok for decades, concerns have been raised regarding inadequate daily dosing and the provision of methadone as a short detoxification program [42]. To conduct an external assessment of methadone treatment programs in Bangkok, 273 IDU who had a history of heroin or other opiate use were recruited in 2009. In total, 52.4% opiate users had accessed methadone treatment in the previous six months, but almost all (98.6%) of them relapsed into active drug use while on treatment. As shown in Figure 2, injecting midazolam twice or more per week was independently associated with being enrolled in methadone treatment (AOR = 1.85; 95% CI: 1.04 – 3.29). High rates of ongoing drug use among methadone patients is likely indicative of the suboptimal system of methadone provision in Bangkok, consistent with previous reports [42]. Moreover, 72.7% reported having stopped methadone treatment, and the most common reason for stopping methadone was incarceration [28].

The second study described the prevalence of and factors associated with HIV testing behavior and explored the willingness to access rapid HIV testing at the MSHRC among HIV-negative IDU or IDU of unknown HIV serostatus. Although 76.2% of the study sample received HIV testing in the previous six months, 56.9% of those who had not been tested in the previous six months reported engaging in HIV risk behavior in the previous six months. Also, it was unclear whether the high rate of testing observed in the study was partially a reflection of the existence of the tenofovir trial (a pre-exposure prophylaxis trial conducted out of Bangkok Metropolitan Administration methadone clinics) [43], as enrolment in the trial was strongly associated with receiving HIV testing (odds ratio = 44.81; 95% CI: 13.44 – 149.45). In total, 74.2% of participants expressed willingness to receive rapid HIV testing at the MSHRC if it were made available [29].

The third study examined access to testing for hepatitis C virus (HCV) among IDU who were living with HIV. Although HIV and HCV co-infection is highly prevalent among Thai IDU [44], and WHO recommends that all people living with HIV be screened for HCV [45], only half (52.2%) of the HIV-infected IDU who enrolled in the study had ever been tested for HCV. This appeared to be related to a lack of awareness of HCV, as primary reasons given for not getting tested for HCV included “never heard of HCV” (65.6%) and “not aware of HCV risks” (37.5%). Further, rates of HCV risk behavior (i.e., syringe sharing) were high (38.2%) among HIV-positive IDU who did not know their HCV status [30]. The deficit in HCV case finding uncovered in this study is significant, given the high rates of HCV risk behavior observed among the study sample and the independent contribution of HCV to morbidity and mortality among HIV-infected IDU [46].

Lastly, an evaluation of the MSHRC was conducted by examining factors associated with access to the MSHRC. In 2008, 29.3% of participants surveyed had accessed the MSHRC, and these individuals were four times more likely to have had difficulty accessing sterile syringes than those who had not previously accessed the MSHRC (AOR = 4.05; 95% CI: 1.67 – 9.80). Forms of support most commonly accessed at the MSHRC included sterile syringes (100%), food and a place to rest (83.8%), and information about HIV (75.7%) and safer injecting (66.2%) [31]. Consistent with a large body of research indicating the benefits of peer-based approaches in providing public health education and services to IDU [47-50], the results of this study demonstrated that the peer-run MSHRC is helping to expand harm reduction programming in Bangkok by reaching a sub-population of IDU at heightened risk of HIV infection.

## Discussion

The results of the Mitsampan Community Research Project reveal that IDU in Bangkok continue to engage in high rates of HIV risk behavior (i.e., syringe sharing) and suffer from high rates of drug-related harm, including overdose. The findings also indicate a lack of appropriate healthcare service provision and problems with suboptimal service delivery among this population, as low rates of sterile syringe access, HCV testing and awareness, and poor outcomes from methadone treatment persist. A number of social and structural factors appear to be driving these problems, including the limited availability of evidence-based interventions targeting IDU, an overreliance on drug law enforcement and incarceration, and a failure to adhere to international guidelines on HIV prevention and care, and drug treatment for IDU. Although Thailand has continued to rely on intensive drug law enforcement, the evidence derived from this study suggest that this approach is failing to produce reductions in drug use, and is associated with human rights violations and the perpetuation of a system of compulsory drug detention that appears to have little impact on the drug use behaviors of IDU in Bangkok. On the other hand, a promising means to reduce drug-related harm among this population was identified at the drug user-run harm reduction center where IDU experiencing difficulty in accessing sterile syringes obtain sterile syringes and other supports.

Many of our findings are congruent with previous studies identifying various pathways through which intensified drug law enforcement produces harmful impacts on the health of IDU and public health [41,51-54]. For example, in previous studies from other countries, increasing policing in drug markets has been shown to have had no effect in reducing injection drug use [54] but instead have resulted in shifts in local drug use patterns and greater harm [51,55]. Our research also demonstrated that perceived increases in police presence in drug markets did not seem to reduce injecting behavior among IDU in Bangkok [27]. As well, data from this project suggested that midazolam and methamphetamine injection is increasing despite ongoing intensive police crackdowns, and injection of these substances was shown to be associated with risk factors of HIV infection and overdose [20,22]. In particular, the finding that the observed prevalence of methamphetamine injection appeared to be higher than the previously reported rates may reflect the concurrent growth of the methamphetamine market suggested in the government’s reports [3], although this explanation warrants a further examination. Further, the observed high rates of police abuse and the strong association with risk taking among IDU in this setting [26] is consistent with previous research indicating that aggressive policing practices not only produce direct harm to IDU but also function as pathways to more distal forms of drug-related harm [41,52,56,57].

Our findings also point to the urgent need for the implementation of harm reduction measures within prisons. According to the latest official report, 56.4% of all incarceration events in Thailand are attributable to drug-related charges [58]. Consistent with previous studies [59-61], our study demonstrated alarmingly high rates of HIV risk behavior among IDU in Bangkok who reported a history of incarceration [24]. Moreover, incarceration was also associated with overdose [23] and was reported to be the most common reason for the discontinuation of methadone treatment [28]. The independent association between overdose and incarceration is consistent with evidence from Western settings indicating that incarceration exacerbates the risk of heroin overdose upon release from prisons as a result of reduced tolerance [62]. These findings suggest that incarceration is contributing to the production of a variety of drug-related harms in this setting. Given the ongoing high rates of incarceration of Thai IDU, our findings reinforce the recommendations by WHO and other UN agencies to implement essential harm reduction programs within prisons, including NSPs [53].

The findings of this research also highlight the need to scale up HIV prevention, care and treatment services targeted for IDU in Bangkok. The HIV prevalence among our study sample (17.4%) was high and similar to the 2010 Integrated Biological and Behavioral Surveys data showing that the HIV prevalence was 21.3% (CI: 15.2-26.5) among a sample of 412 IDU in Bangkok [63]. The observed rate of syringe borrowing (30%) [21] was higher than the rates of syringe sharing among IDU in Bangkok reported by two other studies in 2003–2004 (17%) [37] and 2009 (14%) [64], although the potential differences in sample characteristics make the comparison difficult. The finding that frequent methamphetamine injection had an independent relationship with syringe sharing [22] builds on previous studies showing heightened risk of HIV seroconversion among methamphetamine injectors [36,61] and calls for the scale-up of NSPs for this sub-population of IDU. Although the Thai government supports some harm reduction programs, the illegality of NSPs is still debated [10]. Given the demonstrated strengths of the MSHRC in reaching a sub-population of IDU at heightened risk of HIV infection due to difficulty accessing sterile syringes [31] and the fact that the MSHRC, like many other drop-in centers implementing NSPs that are operated by civil society organizations, relies on the GFATM grant [11], such peer-run interventions should be supported and scaled up by the government. Also, given the substantial level of willingness to access rapid HIV testing at the MSHRC among HIV-negative IDU or IDU of unknown HIV serostatus [29], it may worth exploring the expansion of peer-led harm reduction interventions in this setting, such as the provision of HIV testing and counseling by peers.

The work described in this report has limitations, and the limitations of each individual study are described in detail in the published versions of the studies. First, as in any research that is based on surveying methods, our research cannot prove causal relationships. Second, as the study sample was not randomly recruited, our findings may not be generalizable to the IDU populations in Bangkok or other parts of Thailand. Third, the self-reported data may be affected by response biases, including socially desirable reporting and recall bias. Therefore, we may have over- or underestimated the true prevalence of drug use or HIV risk behaviors among IDU in Bangkok. However, we also note that this type of data has been commonly utilized in other studies examining drug use patterns and found to be valid [65,66]. Finally, building on the present research findings, future research should further examine observed associations between various health and social problems and injection drug use, including the relationship between midazolam injection and dosages of methadone.

## Conclusions

The findings from this research project highlight many ongoing health and social problems related to illicit drug use among IDU in Bangkok. They indicate a lack of appropriate healthcare and harm reduction service provision to this population, problems which are likely contributing to ongoing HIV risk-taking and other drug-related harms within this population. These findings also suggest that the emphasis on criminal justice approaches has resulted in human rights violations at the hands of police, and harms associated with compulsory drug detention and incarceration. Collectively, the findings indicate the need for urgent government endorsement, funding and independent evaluation of a comprehensive set of IDU-specific harm reduction and addiction treatment programs in Bangkok.

## Competing interests

The authors declare that they have no competing interests.

## Authors’ contributions

KH drafted the manuscript and incorporated all suggestions from co-authors. All authors made significant contributions to the conception of the analyses, interpretation of the data, and drafting of the manuscript. All authors read and approved the final manuscript.
